# Preparation and Characterization of Tris(trimethylsiloxy)silyl Modified Polyurethane Acrylates and Their Application in Textile Treatment

**DOI:** 10.3390/polym12081629

**Published:** 2020-07-22

**Authors:** Xuecheng Yu, Ying Xiong, Zhen Li, Hongding Tang

**Affiliations:** Engineering Research Center of Organosilicon Compounds & Materials, Ministry of Education, Sauvage Center for Molecular Sciences, College of Chemistry and Molecular Sciences, Wuhan University, Wuhan 430072, China; yuxuecheng1992@whu.edu.cn (X.Y.); yingxiong@whu.edu.cn (Y.X.); lizhen@whu.edu.cn (Z.L.)

**Keywords:** polyurethane acrylate, UV-curing, silicone, hydrophobicity, textile treatment, oil/water separation

## Abstract

Three series of silicone modified polyurethane acrylate (SPUA) prepolymers were prepared from dicyclohexylmethane-4, 4′-diisocyanate (HMDI), PPG1000, triethylene glycol (TEG), 2-hydroxyethyl acrylate (HEA), and multi-hydroxyalkyl silicone (MI-III) with tris(trimethylsiloxy)silyl propyl side groups. Their structures were confirmed by ^1^H NMR, ^13^C NMR, and Fourier transformed infrared (FTIR) analysis, and SPUA films were obtained by UV curing. The properties of films were investigated by attenuated total reflection (ATR)-FTIR, scanning electron microscopy (SEM), energy dispersive X-ray spectroscopy (EDS), water contact angle (WCA), thermogravimetric analysis (TGA), differential scanning calorimeter (DSC), water and hexane resistance, and tensile testing. The results showed that the structures and dosages of MI-III could influence the polymerization properties, surface properties, water and n-hexane resistance, and thermal and tensile properties of SPUA. For instance, the surface aggregation of tris(trimethylsiloxy)silyl propyl groups (even ~2.5 wt%) could endow SPUA films with less microphase separation, good hydrophobicity, lipophilicity, thermal stability, and mechanical properties. Interestingly, obvious regular winkles appeared on the surfaces of SPUAIII films, which are characterized by relatively high WCA values. However, relatively smooth were observed on the surfaces of SPUAIII films, which also exhibit lower water absorption ratio values. Furthermore, the ordinary cotton textiles would be transformed into hydrophobic and oleophilic textiles after treating with SPUA simply, and they were used in the oil/water separation study. Among them, consistent with water and hexane resistance analysis of SPUA films, SPUAII treated cotton textiles are characterized by relatively small liquid absorption capacity (LAC) values. Thus, phenyl groups and side-chain tris(trimethylsiloxy)silyl propyl groups are helpful to improve the hydrophobicity and lipophilicity of SPUA films. SPUAII-5 (even with 5 wt% MII) treated cotton textiles could efficiently separate the oil/water mixture, such as n-hexane, cyclohexane, or methylbenzene with water. Thus, this material has great potential in the application of hydrophobic treatment, oil/water separation, and industrial sewage emissions, among others.

## 1. Introduction

The water repellents of textiles are widely used in hydrophobic surface treatment, oil/water separation and textile finishing, among others [1,2]. Their hydrophobicity is usually achieved by using fluorine compounds [3], silicone compounds [4], and aliphatic compounds [5]. Among them, fluorine compounds are most widely used because of their ultralow surface energy. However, their practical applications are restricted by the poor low temperature resistance, high cost, and potential biological toxicity [6]. Meanwhile, silicone materials are well known not only for the low surface energy and hydrophobicity, but also for their excellent resistance to high and low temperatures, flexibility, good biocompatibility, and chemical inertness owing to the unique molecular structures [7,8]. Therefore, they have recently been applied to textile hydrophobic treatments [9,10,11]. However, the poor adhesion to the textile and poor mechanical properties of unmodified silicone materials still have to be improved [12].

Polyurethane (PU) is also widely used in textile treatment with many advantages such as durability and wrinkle resistance [13,14]. Compared with traditional moisture-sensitive PU, polyurethane acrylate (PUA), a category of photo-curable resin, not only exhibits good solubility, flexibility and mechanical properties, but also displays better storage stability, water resistance, solvent resistance, and lower melting viscosity [15,16,17]. Moreover, silicone modified polyurethane acrylate (SPUA) would be expected to combine the advantage of silicone and PUA materials [18,19]. 

**Scheme 1:**
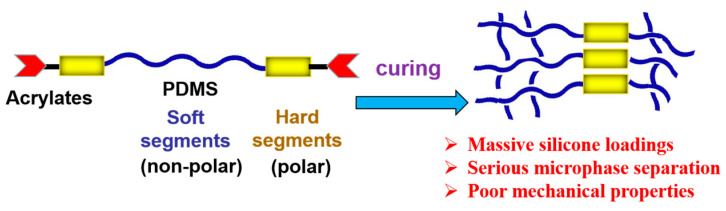
The schematic diagram of main-chain silicone modified polyurethane acrylate (PUA). PDMS, polydimethylsiloxane.

Nowadays, SPUA materials have attracted researchers’ attention to develop their applications of hydrophobic textile treatment [20,21]. In these cases, hydroxyl-terminated polydimethylsiloxane (HO-PDMS-OH) were used to construct the main chains and acted as the hydrophobic ingredients (Scheme 1). Meanwhile, the massive silicone dosages are necessary in order to guarantee sufficient surface aggregation of PDMS segments for high hydrophobicity, as their movements were restricted by the polymer main chains. However, it would lead to serious microphase separation, decreased mechanical properties and high cost, when the ordered hard-segments domains (by hydrogen bonds) distribute in the nonpolar PDMS-segments domains [22,23,24]. In order to keep the balance between hydrophobicity and silicone loading, a workable way is to incorporate silicone into the side chains of SPUA.

**Scheme 2:**
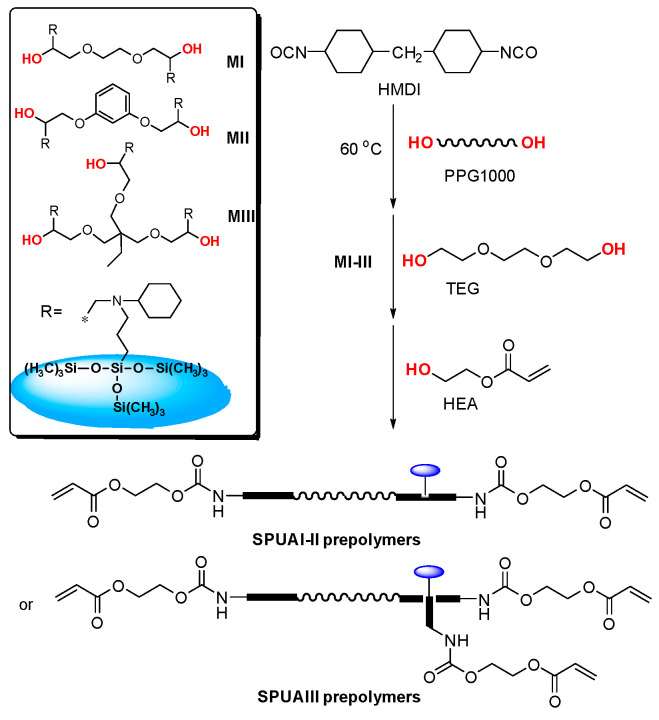
Synthesis of silicone modified polyurethane acrylate (SPUA) prepolymers. MI, multi-hydroxylalkyl silicones; TEG, triethylene glycol; PPG1000, polypropylene glycol; HEA, 2-hydroxyethyl acrylate; HMDI, dicyclohexylmethane-4, 4′-diisocyanate.

**Scheme 3:**
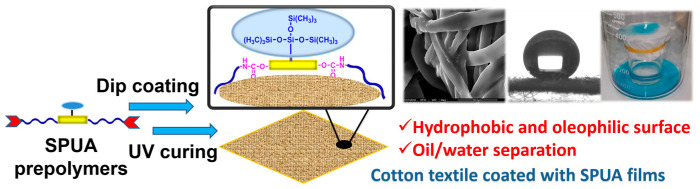
The schematic illustration for the SPUA-treated cotton textiles.

From our previous research [25], side-chain tris(trimethylsiloxy)silyl propyl groups have been demonstrated to exhibit a definite surface migration trend in polyurethane films, which resulted in improved surface hydrophobicity and water resistance with only small silicone loadings. Following our continuing interesting, herein, three series of SPUA prepolymers (Scheme 2) with different silicone structures and contents were prepared from multi-hydroxylalkyl silicones (MI-III), triethylene glycol (TEG), polypropylene glycol (PPG1000), 2-hydroxyethyl acrylate (HEA), and dicyclohexylmethane-4, 4′-diisocyanate (HMDI). Then, SPUA films were obtained by the UV-radiation process. As expected, the SPUA films were characterized by good hydrophobicity. For instance, only 5 wt% of MI loading could change the WCA of the hydrophilic PUA from 79.9° to 110.0°, and decrease the water absorption from 4.1% to 3.0%. From energy dispersive X-ray spectroscopy (EDS) analysis, the measured Si contents on the surface of cured SPUA films are almost double the calculated theoretical average values, indicating the definite surface migration trend of silicone. Moreover, the structures and dosages of introduce silicone could influence the polymerization properties, surface properties, water and n-hexane resistance, and thermal and tensile properties of SPUA. Interestingly, obvious regular winkles appeared on the surfaces of SPUAIII films, which are characterized by relatively high WCA values. However, relatively smooth surfaces were observed on the surfaces of SPUAII films, which also exhibit lower water absorption ratio values. Then, PUA and SPUA treated cotton textiles could be provided by dip coating and UV curing, and used in the study of oil/water separation (Scheme 3). Among them, SPUAII series (with Ph-groups) show relatively small liquid absorption capacity (LAC) values and are the best in the separation of oil/water mixture, such as n-hexane/water, cyclohexane/water, and methylbenzene/water, among others. Thus, these SPUA prepolymers have great potential applications in the hydrophobic treatment, oil/water separation, and industrial sewage emissions, among others.

## 2. Experimental 

### 2.1. Materials

Polypropylene glycol (PPG1000, Mn~1000 g/mol), 2-hydroxyethyl acrylate (HEA), and 2-hydroxy-2-methylpropionphenone (HMPP, Darocur 1173) were purchased from Sigma-Aldrich Chemical (Yongin City, South Korea). Dibutyltin dilaurate (DBTDL), Ethyl acetate (EA) and triethylene glycol (TEG) were obtained from Sinopharm Chemical Reagent Co., Ltd. (Shanghai, China). Dicyclohexylmethane-4, 4′-diisocyanate (HMDI) was kindly offered free by Wanhua Chemical Group Co., Ltd. (Yantai, China). Ethyl acetate (EA) was purified by standard methods before use. The multi-hydroxylalkyl silicones with the tris(trimethylsiloxy)silyl propyl groups (MI-III) were prepared by the previously reported method [25]. The 100% plain-woven cotton textiles (122 g/m^2^) were purchased from Weifang Qirong Textiles Co., Ltd. (Weifang, China), which was further purified by ultrasonic washing with distilled water and ethanol for 10 min, respectively, and then dried in the oven at 80 °C. All other chemicals and solvents were used without further purification.

### 2.2. Instrumentations

The viscosity of the prepolymers was measured by NDJ-5S rotational viscosimeter (Shanghai Ping Xuan Scientific Instrument Co., LTD., Shanghai, China) in mPa.s at 25 °C. ^1^H NMR and ^13^C NMR spectra were obtained with AVANCE AV-300 (Bruker BioSpin, Billerica, Massachusetts, USA) and recorded in CDCl_3_. The chemical structures of prepolymers were examined using FTIR spectroscopy in the Nicolet IS10 (Thermo Fisher Scientific, Waltham, MA, USA). The spectrums were recorded in the region of 400 to 4000 cm^−1^ at a resolution of 4 cm^−1^. Attenuated total reflection Fourier transformed infrared (ATR-FTIR) spectrums of UV-cured films were also recorded on Nicolet IS10, but with a compatible ATR module and germanium crystal at the incident angle of 45°. The spectra were recorded from 400 to 4000 cm^−1^ at a resolution of 4 cm^−1^ and 64 scans. Thermogravimetric analysis (TGA) was conducted using Setsys16 (Aetaram, France) from 25 to 600 °C at 20 °C/min heating rate in nitrogen atmosphere. Differential scanning calorimeter (DSC) was performed on a DSC822^e^ (Mettler Toledo, Zurich, Switzerland) from 100 to −50 °C at a cooling rate of 10 °C/min in nitrogen. Scanning electron microscopy (SEM) and energy dispersive X-ray spectroscopy (EDS) analysis were carried out using Quanta 200 (FEI NanoPorts, Hillsboro, OR, USA) at an accelerating voltage of 20 kV. The films and textiles were coated with gold to study the surface morphologies. The data of EDS were collected from a scanned region of about 100 × 100 square micrometres. Water contact angle (WCA) was performed on a G10 (KRUSS, Germany) instrument equipped with a camera through the sessile drop method, and the results were recorded one min later. For each sample, at least three measurements were conducted and then the average value was calculated. Tensile tests were performed on SANS-CMT5 105 (Shenzhen Sans Testing Machine Co., LTD., Shenzhen, China) with a crosshead speed of 200 mm/min (L_0_ = 5 mm) at room temperature. Tests of at least three identical samples were conducted and the average value was calculated.

### 2.3. Synthesis of Polyurethane Acrylate (PUA) Prepolymer

In order to contrast with silicone modified counterparts, PUA prepolymer without silicone was prepared through the following procedure. A 250 mL of four-necked flask equipped with a condenser, a dropping funnel, a magnetic stirrer, and a device for argon flow was charged with 13.10 g (0.050 mol) of HMDI, 10.00 g (0.010 mol) of PPG1000, 20.00 g of ethyl acetate, and 0.02 g of DBTDL, and the mixture was stirred at 60 °C for 4 h. Then, the mixture of 4.50 g (0.030 mol) of TEG and 20.00 g of ethyl acetate (EA) was added dropwise at 60 °C within 2 h. After addition, the mixture was reacted at 70 °C for 1 h until the measured content of the residual -NCO groups reached the theoretical value (calculated from the recipe, 6.67 × 10^−4^ mol/g) to provide the –NCO terminated prepolymer. Subsequently, 2.40 g (0.020 mol) of HEA was added into the -NCO terminated prepolymer and the mixture was kept at 60 °C for 4 h until the characteristic FTIR peak at ~2264 cm^−1^ disappeared. This prepolymer was used for the preparation of PUA films without any further purification. FTIR (film, cm^−1^): 3328, 2968, 2927, 2858, 1715, 1637, 1535, 1450, 1374, 1247, 1100, 931, 777. ^1^H NMR (CDCl_3_, 300 MHz) δ [ppm]: 0.7–1.9 (N–CH–C_5_**H**_9_–C**H**_2_–C_5_**H**_9_–CH–N, C**H**_3_–CH–O), 3.1–3.8 (–O–C**H**_2_–C**H**_2_–O, –CH_2_C**H**(CH_3_)O–, N–C**H**–), 4.2–4.4 (–COO–C**H**_2_, –COO–C**H**(CH_3_)–), 4.6–5.0 (–N**H**–CO–O–), 5.7–6.5 (C**H**_2_=C**H**–). ^1^^3^C NMR (CDCl_3_, 75 MHz) δ [ppm]: 17.3, 27.9, 29.6, 32.0, 32.5, 33.5, 46.9, 50.3, 60.7, 63.6, 66.1, 69.7, 72.9, 75.2, 128.1, 131.2, 155.6, 165.9.

### 2.4. Synthesis of Silicone Modified Polyurethane Acrylate (SPUA) Prepolymers

SPUA prepolymers were prepared according to Scheme 1. In order to maintain a consistent molar content of hard segments, the molar number of -OH (TEG and MI-III) is 0.03 mol in all experiments. Meanwhile, the molar number of HMDI or PPG1000 or HEA is consistent.

For clarity, the SPUAI with four different dosages of MI were prepared by the same procedure, but different recipes, which are listed in Table 1. The SPUA prepolymer prepared from MI with 5 wt% silicone is specified as SPUAI-5, and it is used as an example to demonstrate the preparation procedure for all the prepolymers. A 250 mL of four-necked flask equipped with a condenser, a dropping funnel, a magnetic stirrer, and a device for argon flow was charged with 13.10 g (0.050 mol) of HMDI, 10.00 g (0.010 mol) of PPG1000, 20.00 g of EA, and 0.02 g of DBTDL, and the mixture was stirred at 60 °C for 4 h. Then, the mixture of 4.50 g (0.030 mol) of TEG, 1.57 g (0.0015 mol) of MI, and 20.00 g of EA was added dropwise at 60 °C within 2 h. After addition, the mixture was reacted at 70 °C for 1 h until the measured content of the residual -NCO groups reached the theoretical value (calculated from the recipe, 6.38 × 10^−4^ mol/g) to provide the -NCO terminated prepolymer. Subsequently, 2.40 g (0.020 mol) of HEA was added into the -NCO terminated prepolymer and the mixture was kept at 60 °C for 4 h until the characteristic FTIR peak at ~2264 cm^−1^ disappeared. The prepolymer was used for the preparation of SPUA films without any further purification. FTIR (film, cm^−1^): 3330, 2969, 2928, 2857, 1715, 1637, 1535, 1450, 1374, 1249, 1192, 1100, 1050, 844, 757. ^1^H NMR (CDCl_3_, 300 MHz) δ [ppm]: −0.1–0.1 (–Si–C**H**_3_), 0.2–0.5 (Si–C**H**_2_–), 0.7–1.9 (N–CH–C_5_**H**_9_–C**H**_2_–C_5_**H**_9_–CH–N, C**H**_3_–CH–O, N–CH–C_5_**H**_10_), 3.1–3.8 (–O–C**H**_2_–C**H**_2_–O, –CH_2_C**H**(CH_3_)O–, N–C**H**–, N–C**H**_2_–), 4.2–4.4 (–COO–C**H**_2_–, –COO–C**H**(CH_3_)–), 4.6–5.0 (–N**H**–CO–O–), 5.7–6.5 (C**H**_2_=C**H**–). ^13^C NMR (CDCl_3_, 75 MHz) δ [ppm]: 0.0, 15.3, 26.2, 27.8, 30.3, 31.8, 45.3, 48.5, 58.7, 61.8, 64.4, 67.8, 68.7, 71.1, 73.2, 126.3, 129.5, 153.9, 164.2.

### 2.5. Photopolymerization Experiments

The light-induced photopolymerization experiments of PUA or SPUA prepolymers were carried out by real-time infrared spectroscopy (RT-IR). Different contents of HMPP were dissolved in the prepared PUA or SPUA prepolymers to form the photosensitive formulations. Then, they were deposited on KBr pellets in laminate for irradiation with a high-pressure mercury lamp (Ushio SH-250DB, Japan, I = 40 mW/cm^2^). The evolution of the double-bond content was continuously monitored by real time FTIR spectroscopy (Nicolet IS 10, America) at 1650–1610 cm^−1^ [26]. The double-bond conversions (DC) were calculated from Equation (1) [27].

(1) $$\mathrm{DC}\;\left(\%\right)=\left(1-\frac{A_{X,t}\;}{A_{X,0\;}}\times \frac{\;A_{\mathrm{ST},0}}{A_{\mathrm{ST},\;t}}\right)\times 100\%$$

where A_0_ and A_t_ represent the areas of the IR absorption peaks of the functional groups of the sample before and after exposure during time t. The subscripts ST and X represent the internal referential ester carbonyl peak at 1715 cm^−1^ and the double bond peak at 1650–1610 cm^−1^, respectively. For each sample, the series of real-time FTIR runs are repeated at least three times.

### 2.6. Preparation of the UV-Cured Films 

HMPP (1.00 wt %) was dissolved in the prepared PUA and SPUA prepolymers to form a uniform solution, which was then coated on the Teflon plate. After being placed at room temperature for 24 h to remove the solvent (EA), they were exposed for UV curing under a high-pressure mercury lamp (40 mW/cm^2^, Ushio SH-250DB) for 600 s in N_2_ atmosphere.

### 2.7. Water and n-Hexane Absorption of the UV-Cured Films

Water and n-hexane absorption of PU and SPU films were evaluated. The weighted cured films (W_0_) were immersed in the distilled water or n-hexane at room temperature for 24 h, followed by wiping off the surface water or n-hexane with a piece of filter paper, and then tested for weight W_1_. For each sample, the experiments are repeated at least three times. The ratio (A) for absorbed water or n-hexane in the cured film was calculated by Equation (2).

(2) $$A=\frac{\left(W_{1}-W_{0}\right)}{W_{0}}\times 100\%$$

### 2.8. PUA- or SPUA-Treated Cotton Textiles 

Treated cotton textiles could usually be prepared by dip coating and UV curing (as shown in Figure 5A) [28]. Thus, cotton textiles (10 × 10 cm) were first soaked in the solution composed of 2.0 g of the prepared PUA or SPUA prepolymers, 0.02 g of HMPP, and 8.0 g of ethyl acetate for 30 s. After the textiles were taken out and then dried in an 80 °C oven for 10 min, each side of the textiles was UV crosslinked in N_2_ atmosphere under a high-pressure mercury lamp (40 mW/cm^2^, Ushio SH-250DB) for 600 s to provide the PUA- or SPUA-treated textiles.

### 2.9. Liquid Absorption Capacity (LAC) of Untreated and Treated Cotton Textiles 

The liquid absorption capacity (LAC) of textiles could be determined according to standard ISO 9073-6:2003 “Textiles. Test methods for nonwovens. Part 6: Absorption” [29]. Textiles were cut into 10 × 10 cm^2^ samples, and then weighed before and after immersing in water for 60 s. For each sample, the experiments are repeated at least three times. The LAC values were obtained using Equation (3).

(3) $$LAC=\frac{m_{n}-m_{k}}{m_{k}}\times 100\%$$

where *m_n_* is the mass of wet textile (g) and *m_k_* is the mass of dry sample (g).

## 3. Results and Discussion

### 3.1. Synthesis and Characterization of PUA and SPUA Prepolymers

As continuous interest, the multi-hydroxylalkyl compounds with tris(trimethylsiloxy)silyl propyl groups (MI-III) were used to construct photo-sensitive SPUA. Three series of SPUA prepolymers with different silicone contents were prepared. For comparison, PUA without silicone incorporated was also synthesized. In their structures (Scheme 1), polypropylene glycol (PPG1000) acts as the soft segments in polymers, and MI-III, TEG, and HMDI act as the hard segments. Meanwhile, the molar number of HMDI or PPG1000 or HEA is consistent in all series, which means the molar content of soft segments or hard segments is mainly consistent.

**Figure 1:**
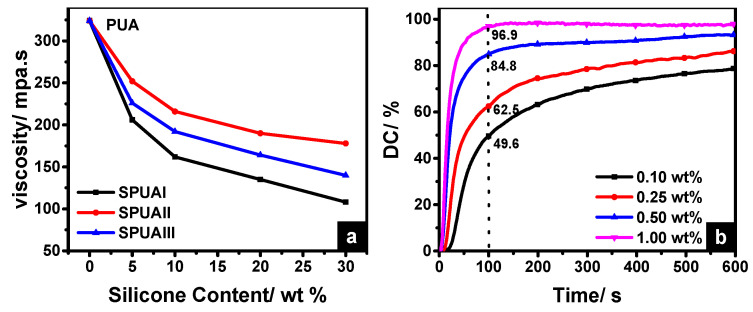

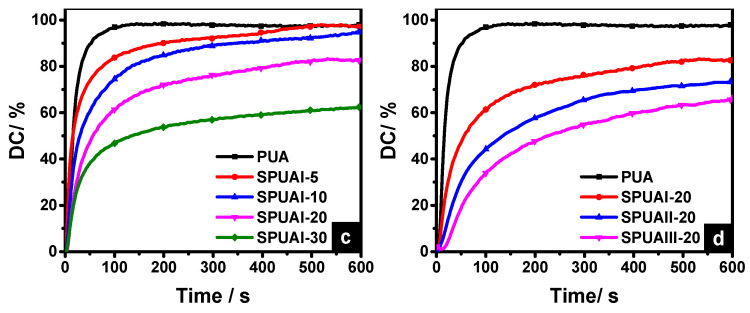
(**a**) Viscosity values of polyurethane acrylate (PUA) and silicone modified PUA (SPUA) prepolymers. Photopolymerization profiles of (**b**) PUA prepolymers initiated by 2-hydroxy-2-methylpropionphenone (HMPP) with different contents, (**c**) PUA and SPUAI prepolymers initiated by 1.00 wt% of HMPP, and (**d**) PUA and SPUA prepolymers initiated by 1.00 wt% of HMPP. DC, double bond conversion.

During the preparing process, the obvious viscosity differences between PUA and SPUA prepolymers were observed and the measured values are shown in Figure 1a. The viscosity of prepolymers could be affected by many factors, such as temperature, molecular weight, inter-chain force, crosslinking density, and solvent [30]. As can be seen, the viscosity of SPUAI-5 prepolymer (206 mPa.S) is much lower than that of PUA prepolymer (324 mPa.S). In contrast to PUA prepolymer, the loadings of solvents (EA) in all prepolymers are consistent (40.00 g), and the main-chain chemical structures of their prepolymers are maintained. Thus, only 5 wt% of silicone (~2.5 wt% side-chain tris(trimethylsiloxy)silyl propyl groups) could decrease the viscosity of SPUA prepolymers distinctly. The main reason is that the low-polarity side-chain silicone in SPUA decreases the formation of the inter-chain hydrogen bonds. The low viscosity should also be useful in practical applications as it could reduce the usage of volatile organic compounds (VOCs). However, the rigid phenyl groups in the SPUAII main chains and the higher crosslinking density in SPUAIII bring about the higher viscosities in comparison with the SPUAI prepolymers.

The NMR and FTIR spectra of PUA and SPUAI-5 are exampled to demonstrate the structures of the prepolymers. As can be seen from the NMR spectra of PUA prepolymer (ESI Figures S2 and S15) and SPUAI-5 prepolymer (ESI Figures S3 and S16), the peaks at 4.8 and 5.0 ppm in ^1^H NMR spectra and 156 ppm in ^13^C NMR spectra are ascribed to the protons and carbon in carbamate/urethane groups (–NH–CO–O–), demonstrating the reactions between –NCO and –OH groups [31]. The measured –NCO content reached the theoretical value of the prepolymer before adding HEA, showing the complete reactions between isocyanates and polyols. The peaks at 5.8, 6.1, and 6.4 ppm in ^1^H NMR spectra and 166, 131, and 128 ppm in ^13^C NMR spectra result from the introduction of acrylate groups (–O–CO–CH=CH_2_). Meanwhile, the peaks at 0.0 ppm in ^1^H and ^13^C NMR spectra of SPUAI-5 result from Si-CH_3_. In the FTIR spectra of PUA and SPUAI-5 prepolymers (ESI Figure S1a), the peaks at around 3330 cm^−1^(N–H), 1715 cm^−1^ (C=O in acrylate) and 1535 cm^−1^ (N–H and N–C(O) in carbamate groups) and the disappeared characteristic absorption peak of -NCO (~2264 cm^−1^) confirm that the reaction between isocyanates and HEA is complete. Meanwhile the characteristic peaks of C=C–CO groups have been observed at about 1637 cm^−1^ [26]. Additionally, the peaks of about 844 and 757 cm^−1^ show the presence of Si–(CH_3_)_3_ in SPUAI-5 spectrum. Thus, the prepolymers of PUA and SPUAI-5 were synthesized successfully.

### 3.2. Photopolymerization Kinetics of PUA and SPUA Prepolymers

In order to investigate the photopolymerization behaviors of PUA and SPUA prepolymers, real-time FTIR spectroscopy was used to monitor the double bond conversion (DC) under UV irradiation (Ushio SH-250DB, 40 mW/cm^2^). A widely used commercial UV photo-initiator, HMPP, was used for photopolymerization. The impacts of HMPP contents to the photopolymerization behaviors of PUA prepolymers were investigated and the results are shown in Figure 1b. As can be seen, an obvious oxygen inhibition was observed when HMPP content is as low as 0.25 wt%. With the increase of HMPP content, the degree of double-bond conversion (DC) and the rate of polymerization were improved. The DC could reach as high as 96.9% at 100 s when the content of HMPP arrived at 1.00 wt%. Thus, 1.00 wt% of HMPP content was also used in the photopolymerization investigation in the following SPUA prepolymers. In the photopolymerization profiles of SPUAI prepolymers (Figure 1c), however, it was found that the DC decreased with the increase of the silicone content. The side-chain silicone groups are supposed to restrict the diffusion and mobility of terminal double bonds, indicating that uncured double bonds would be trapped in the polymeric networks [32]. Moreover, the decreased mass fraction of double bonds with the continuous increased silicone content would also lead to a lower DC value [33,34].

Furthermore, compared with SPUAII or SPUAIII series, SPUAI series exhibit a higher DC value (Figure 1d). The possible reason is that the rigid phenyl linking groups in SPUAII and the increased crosslinking density in SPUAIII make the main-chain movements harder than that of SPUAI [35], which is consistent with their viscosity differences. On the other hand, the crosslinking density of SPUAI-II decreases with the increase of silicone content. Therefore, high acrylate content and easy movement of polymer segments are helpful to obtain a high DC value in SPUA.

### 3.3. Properties of PUA and SPUA Films

**Figure 2:**
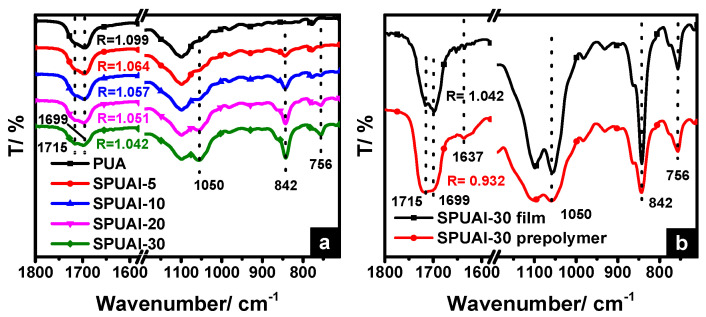
(**a**) Attenuated total reflection Fourier transformed infrared (ATR-FTIR) spectra for the PUA and SPUAI films, (**b**) ATR- FTIR spectrum of SPUAI-30 film and FTIR spectrum of its prepolymer.

ATR-FTIR is an efficient method to investigate the surface properties of polymer films. The ATR-FTIR spectra of PUA and SPUAI series of films are taken as examples and the results are presented in Figure 2a. From that, the intensity of absorption peaks at 842 and 756 cm^−1^ (Si–(CH_3_)_3_) and 1050 cm^−1^ (Si–O–Si) would increase, accompanied by the increase of silicone contents. Moreover, the hydrogen bonded carbonyl groups appear at 1699 cm^−1^, and the value is slightly lower than that of the free PU carbonyl groups (~1715 cm^−1^) [36]. The carbonyl hydrogen bonding index (R) could be obtained by A_1699_/A_1715_ to describe the degree of the carbonyl groups participating in hydrogen bonding [37]. It could be found that R would decrease with the increase of silicone contents, meaning the decrease of inter-chain hydrogen bonds and ordered hard segments in their films [38].

As can be seen from Figure 2b, the absorption peaks at around 1637 cm^−1^ for –CH=CH_2_ in acrylates in SPUAI-30 prepolymer almost disappeared after photopolymerization, demonstrating the completion of UV curing on the film surfaces. Compared with the prepolymer, the film is characterized by a bigger R value (1.043), meaning the formation of hydrogen bonds during the UV-curing process.

For the further investigation, SEM and EDS analysis were used to evaluate the surface morphologies and the elemental contents of PUA and SPUA films. From the results in Table 2, a tiny amount of Si could also be measured in the pure PUA sample. The reason is that background noise peaks were calculated into the content of Si, and the relative error limit of EDS analysis would be very big when the content of measured element content <1.0 wt%. Compared with the calculated average Si contents, the measured Si contents of SPUA surfaces are much higher. This should result from the polymer–air interface migration of silicone groups with low surface energy [39,40]. The silicon enrichment factor (F) can be obtained by the measured Si content divided by the calculated one to evaluate the surface migration trend of silicone, and a high F value demonstrates the high enriching efficiency [41]. As can be seen in Table 2, the F values of SPUAI and SPUAII are higher than those of SPUAIII with same silicone content, which could be explained in that the side-chain silicone in tri-functional MIII are restricted to the junction of main chains and hindered to migrate to the surface. The F values in SPUA decrease with the increase of silicone contents, implying that lower loading of silicone exhibits the stronger thermodynamic driving trend for surface migration [42,43].

**Figure 3:**
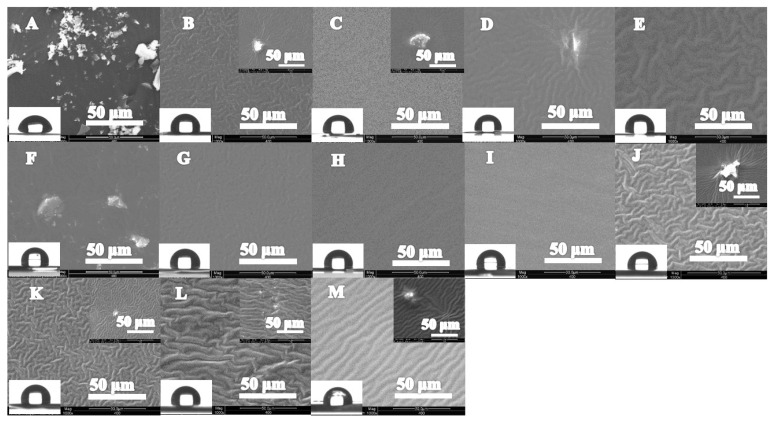
The representative scanning electron microscopy (SEM) images of PUA and SPUA films: (**A**) PUA, (**B**) SPUAI-5, (**C**) SPUAI-10, (**D**) SPUAI-20, (**E**) SPUAI-30, (**F**) SPUAII-5, (**G**) SPUAII-10, (**H**) SPUAII-20, (**I**) SPUAII-30, (**J**) SPUAIII-5, (**K**) SPUAIII-10, (**L**) SPUAIII-20, and (**M**) SPUAIII-30 (bottom left insets are the pictures of water contact angle (WCA) measurements and upper right insets are the pictures of microphase separation).

The surface SEM images of PUA and SPUA films are shown in Figure 3. The PUA (Figure 2a) film shows a typical microphase separation morphology, in which many protrusions of hard-segment domains have dispersed in the soft segments [44]. Incorporation of only 5 wt % MI or MII into PUA could significantly decrease the microphase separation (Figure 3B,F), evidenced by the fact that only a little bit of agglomerations could be observed (inset). Meanwhile, the protrusions become more and more rare with the increase of silicone loadings (Figure 3B–I). The reason should be that the low polar side-chain silicone groups could efficiently decrease the formation of hydrogen bonds, which hinders the agglomeration of hard segments. Among three series, SPUII series (Figure 3F–I) display the smoothest surfaces with the least microphase separation, as the phenyl linking groups in MII are helpful to the improve the compatibility of different segments in SPUA.

Interestingly, regular wrinkles appear in SPUAI series (Figure 3D,E), and they are more obvious in SPUAIII series. However, almost no wrinkles are observed in PUA (the most serious microphase separation) and SPUAII (the less microphase separation) films. Thus, the degree of microphase separation could be an impact factor for the formation of this kind of wrinkles. On the other hand, the increased crosslinking, resulting from the photo-crosslinking and the introduction of trihydroxyalkyl MIII, brings about the volume shrinkage [45], which provides the force to form wrinkles. Therefore, the structure and loading of the silicone and the crosslinking density provide opportunities to adjust the surface morphology of SPUA films

Surface water–air contact angle is usually used to evaluate the hydrophobicity of material surface. According to Cassier theory and Wenzel model theory, the water contact angle (WCA) is determined by the surface chemical composition (intrinsic nature of hydrophobicity) and the surface geometrical microstructures (roughness) [46]. The WCA data of PUA and SPUA films are summarized in Table 2. The WCA of PUA film is measured to be 79.9°, implying its hydrophilic surface. The WCA of SPUAI-5 film increases swiftly to 110.1° with only 5 wt% MI loading (~2.5 wt% side-chain tris(trimethylsiloxy)silyl propyl groups), mainly resulting from the efficient surface migration of hydrophobic silicone side groups. However, a further increase in MI loading to 10 wt% brings about 9° decrease in WCA for SPUAI-10 (101.1°), mainly owing to a smoother surface. Furthermore, a slightly increased WCA in SPUAI-20 (103.3°) or SPUAI-30 (103.4°) is observed, further demonstrating that the micro roughness of film surface is also an important factor to the WCA [47,48]. Therefore, SPUAI-5 (Figure 3B), SPUAII-5 (Figure 3F), and SPUAIII series (Figure 3J–M) have relatively rougher surfaces and exhibit relatively higher WCA values among SPUA films.

The thermal stability of PUA is also an important property for practical applications. Generally, its thermal stability depends on several factors such as structure, composition, crosslinking density, molecular weight, and inter-molecular forces [49,50,51]. The thermal stabilities of the PUA and SPUA films were investigated by TGA under N_2_ atmosphere (Figure S34), and the results are listed in Table 2. It is easy to find that the values of T_on_ (the temperature of 5% weight loss) for the SPUAI and SPUAII with low silicone contents (such as lower than 20 wt%) are higher than that of PUA. In these cases, their main chains are almost the same, which means that the low loading of side chain tris(trimethylsiloxy)silyl propyl groups in PUA could increase its thermal stability. However, we also find that excessive silicone loadings (such as 30 wt%) would decrease the thermal stability of SPUA, which should mainly result from the lower crosslinking density owing to the decreased content of acrylate and the lower DC values with the increase of silicone content, as mentioned before. It is noticed that the values of T_50_ for SPUA films are bigger than that of PUA, meaning that introduced silicone could enhance the thermal stability in a relatively high temperature and expand the application temperature range [52]. This may result from the protective char layer containing silica formed by thermal decomposed silicone [53]. Furthermore, compared with SPUAI series, SPUAII and SPUAIII series exhibit better thermal stabilities, which result from the phenyl groups incorporated in hard segments in SPUAII series and the increased crosslinking density in SPUAIII series.

The glass transition temperature (T_g_) of PUA and SPUA films was obtained from DSC (Table 2). Generally, T_g_ of polymers could be influenced by many factors, such as hydrogen bonds, soft and hard segments, the polymer chain motion ability, phase separation, cross-linking density, and so on [54]. From Table 2, SPUAI and SPUAII films exhibit lower T_g_ values than that of PUA, and the T_g_ values would decrease with the increase of silicone content. This should be attributed to the decreased hydrogen bonds caused by side-chain silicone groups [55], and the lower crosslinking density [56]. Among three SPUA series, the T_g_ values of SPUAII and SPUAIII series are higher than those of SPUAI series, showing that increased rigid architecture and crosslinking density would restrict the motion of polymer chains and increase T_g_ [57].

**Figure 4:**
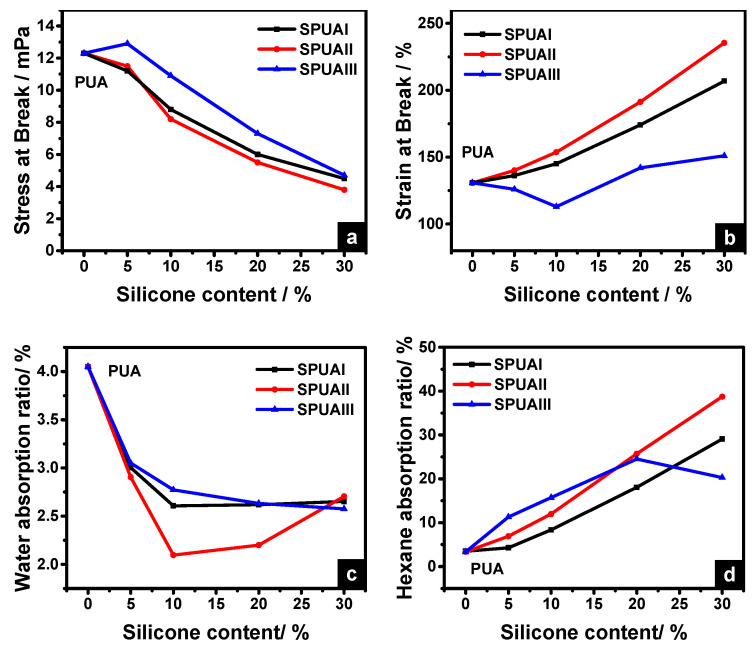
The (**a**) stress and (**b**) strain at break and (**c**) water and (**d**) n-hexane absorption ratio of PUA and SPUA films.

The mechanical properties of PUA and SPUA films are characterized by the strain and stress at break. As shown in Figure 4a,b, the stress at break of SPUAI and SPUAII films decreases with the increase of silicone content (Figure 4a), accompanied by a corresponding increase in strain at break (Figure 4b). In comparison with PUA film, SPUAI-5 and SPUAII-5 films show slightly lower stress at break (Figure 4a). However, the further increase of the silicone content brings about the swiftly decreased stress at break.

The slight decrease of stress at break in SPUAI-5 and SPUAII-5 films should mainly result from the side-chain silicone groups, which decrease the formation of the hydrogen bonds [58]. Moreover, the less hydrogen bonds and lower crosslinking density lead to the swiftly decreased tensile strength and increased elongation at break with the increase of silicone content [59,60]. As aforementioned, the SPUAIII series possess extra increased crosslinking density by the introduction of tri-functional MIII. Therefore, SPUAIII series display relatively high tensile stress and low strain at break among three series.

In order to evaluate the water and hexane resistance of PUA and SPUA films, their water and n-hexane absorption ratios are summarized in Figure 4c,d. As seen from Figure 4c, the water absorption ratios of SPUAI and SPUAII series decrease at first, and then increase with the increase of the silicone contents, and the smallest value is obtained when the silicone content is 10 wt% in each series. As mentioned before, the crosslinking density of SPUAI-II series would decrease with the increase of silicone content, which should lead to higher water absorption of films. Meanwhile, a high silicone content causes the more hydrophobic surface and brings about lower water absorption [61]. Therefore, the hydrophobic silicone plays a leading role in water resistance when the silicone content is lower than 10 wt%. With the further increased silicone content, the decreased crosslinking density plays a leading role. Furthermore, the lowest water absorption ratio of SPUII-10 in three series of SPUA implies that the phenyl linking groups are helpful to improve water resistance.

However, the n-hexane absorption ratios of SPUAI-II series increase with the increased silicone content, which should be attributed to the increased low polar silicone groups and the decreased crosslinking density. Interestingly, the hexane absorption ratio of SPUAII series is higher than that of SPUAI series accordingly, which should result from the oleophilic phenyl groups and lower crosslinking density of SPUAII series than that of SPUAI series.

For SPUAIII series, the water absorption continuously decreases with the increase of MIII content, resulting from the continuously increased silicone loading and crosslinking density. Among SPUAI-III series, the highest water absorption ratios of SPUAIII series are observed when the silicone content is lower than 20 wt%. This should be because the surface silicone contents of SPUAIII films are lower than those of SPUAI and SPUII films from EDS analysis. With the further increased silicone loadings, crosslinking density plays a leading role and SPUIII-30 give the lowest value compared with SPUI-30 and SPUII-30. However, the n-hexane absorption ratios of SPUAIII series first increase and then decrease with the increase of silicone content, in which SPUAIII-30 silicone content showed a lower absorption ratio because the higher crosslinking density plays a more important role.

### 3.4. SPUA-Treated Cotton Textiles 

**Figure 5:**
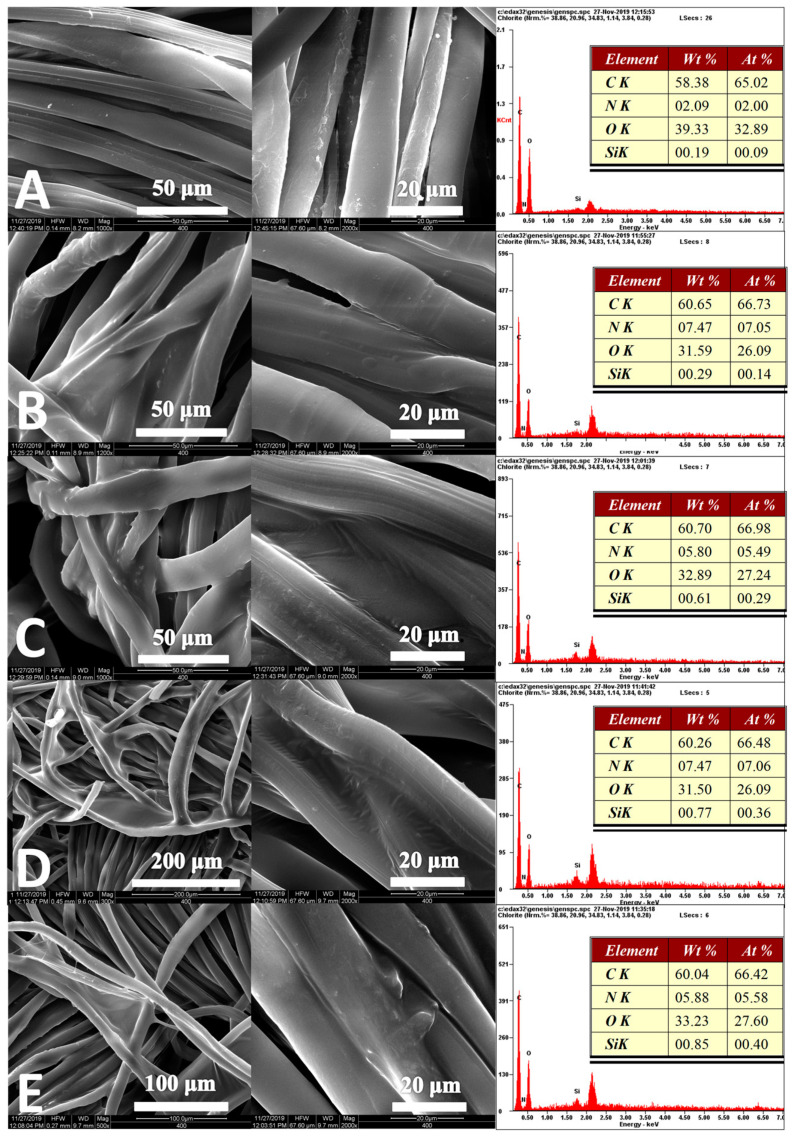
The representative SEM and EDS images of the (**A**) cotton textiles and the cotton textiles treated with (**B**) PUA, (**C**) SPUAI-5, (**D**) SPUAII-5, and (**E**) SPUAIII-5.

As aforementioned, SPUA films exhibit distinct water resistance and surface hydrophobicity. Therefore, their applications in surface hydrophobic treatment of cotton textiles were carried out. The treated cotton textiles could be obtained by dip coating and UV curing, and the WCA data of them are listed in Table 2. The WCA of the cotton textile treated with PUA (109.7°) is much higher than that of the hydrophilic PUA film (79.9°), mainly because of the surface roughness of cotton textiles (Figure 5B) [62]. Moreover, the WCAs of cotton textiles treated with SPUA are as high as ~130°. As can be seen from Figure 5B–E, the SEM image of the cotton textile treated with SPUA shows that the cotton fibres are coated with polymer films evenly and sufficiently. This kind of cover could not only bring about the hydrophobic silicone groups aggregated on the surface of coated SPUA films (Figure 5B–E), but also maintain the micro-roughness of cotton textiles [62].

**Figure 6:**
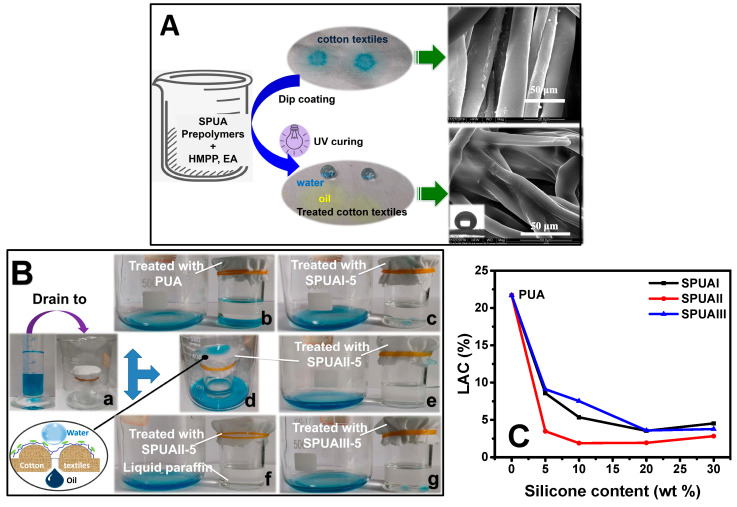
(**A**) Schematic illustration of the cotton textiles treatment process. (**B**) The device images for the oil/water separation process (**a**). The results of the n-hexane/water mixture separated with the treated cotton textiles by PUA (**b**), SPUAI-5 (**c**), SPUAII-5 (**e**), and SPUAIII-5 (**g**). The separation result of the 10th repeated separation of the n-hexane/water mixture with SPUAII-5-treated cotton textiles (**d**). The result of liquid paraffin–water mixture separated with SPUAII-5-treated cotton textiles (**f**). (**C**) The liquid absorption capacity (LAC) results for PUA- and SPUA-treated cotton textiles (the water for oil/water separation experiments were dyed with methylene blue).

However, the n-hexane droplets (dyed yellow in Figure 6A) could completely wet the treated cotton textiles. That is to say, SPUA-treated cotton textiles show the hydrophobic and oleophilic properties, and may have potential application in oil/water separation. Thus, the oil/water separation experiments of the treated cotton textiles were carried out. As a model device for oil/water separation (Figure 6B(a)), the treated cotton textile was covered on a small beaker, which was placed in a bigger beaker [12]. If the oil could penetrate through the treated textile, but the water could not, this apparatus could separate oil/water effectively. Then, the mixture of n-hexane and the dyed water with methylene blue was drained slowly along a glass rod onto the covered cotton textile on the small beaker. Lots of water accompanied by n-hexane penetrated into the small beaker (Figure 6B(b)) when PUA-treated cotton textile was used. In the cases of SPUAI-5- and SPUAIII-5-treated cotton textiles, only a little bit of water leaked into the small beaker (Figure 6B(c,g)). Moreover, no water went through the SPUAII-5-treated cotton textile (Figure 6B(e)), but all of n-hexane penetrated through it. Thus, it could realize the oil/water separation effectively. Furthermore, the wettability of treated cotton textiles was investigated by LAC (Figure 6C). The bigger LAC value of PUA-treated cotton textile should result from the strong polar groups [63]. Consistent with previous water and hexane resistance analysis of SPUA films, SPUAII-treated cotton textiles are characterized by relatively small LAC values (Figure 6C). The possible reason is that oleophilic phenyl groups and nonpolar silicone groups are helpful for the water resistance and the passage of oil, as mentioned before.

In order to evaluate its durability and reusability, the SPUAII-5-treated cotton textile was used to separate n-hexane/water mixture repeatedly only with a drying process in 80 °C in the oven for 10 min after each separation. The result shows that it still displays the good n-hexane/water separation property (Figure 6B(d)) after ten cycles. SPUAII-5-treated cotton textiles can also efficiently separate other oil/water mixtures such as cyclohexane/water and methylbenzene/water mixtures. However, it was found that the treated textile could not efficiently separate the liquid paraffin/water mixture (Figure 6B(f)), as the liquid paraffin has a relatively high viscosity. Therefore, this kind of treated cotton textiles is suitable to separate the mixture of water and some oils with low viscosities.

## 4. Conclusions

In summary, three series of SPUA prepolymers with different silicone contents were synthesized successfully. The tris(trimethylsiloxy)silyl propyl side chains result in the decreased viscosity in SPUA prepolymers, implying less volatile organic solvents needed in future application for economic purpose. Then, the SPUA films were provided by the UV irradiation process.

To assess the properties influenced by the structures and dosages of silicone (MI-III), RT-IR, ATR-FTIR, SEM, EDS, WCA, TGA, DSC, water and hexane resistance, tensile testing, and LAC measurements were performed. The RT-IR results showed that a high content of acrylates and easy movement of polymer chains are helpful to obtain high DC values of SPUA. ATR-FTIR, DSC, and SEM demonstrated that tris(trimethylsiloxy)silyl propyl side chains could restrict the formation of hydrogen bonds, which would decrease the T_g_ values and microphase separation degree of SPUA films. Moreover, relatively smooth surfaces were observed on the surfaces of SPUAII films, implying that phenyl groups have good compatibility with other segments. Obvious regular winkles also appeared on the surfaces of SPUAIII films (with tri-functional MIII), which are characterized by relatively high WCA values. From EDS analysis, the definite surface aggregation of side-chain silicone groups could bring about sufficient surface silicone segments with low silicone loadings. As expected, WCA and water and hexane resistance results implied the significantly improved hydrophobicity and lipophilicity of SPUA films (even containing 5 wt% silicone). From TGA and tensile testing, SPUA films with low silicone content are characterized by improved thermal stability and maintained mechanical properties. Noticeably, phenyl groups are helpful to improve the water resistance, tensile strength, and thermal stability from TGA and tensile testing of SPUA films.

Furthermore, their applications in surface hydrophobic treatment of cotton textiles were investigated. It was shown that cotton fibres could be coated with SPUA films evenly and sufficiently by dip coating and UV curing. Moreover, phenyl groups and side-chain tris(trimethylsiloxy)silyl propyl groups are helpful to improve the hydrophobicity and lipophilicity of SPUA films. LAC analysis of treated cotton textiles, which is connected both with surface roughness and their chemical composition, indicated that relatively small LAC values of SPUAII treated cotton textiles should result from oleophilic phenyl groups and nonpolar silicone groups (consistent with previous water and hexane resistance analysis of SPUA films). Thus, SPUAII-5 (even with 5 wt% MII)-treated cotton textiles can also efficiently separate oil/water mixtures such as cyclohexane/water and methylbenzene/water mixtures. Thus, it is shown that high crosslinking density, phenyl groups, and side-chain tris(trimethylsiloxy)silyl propyl groups are good for constructing hydrophobic treatment agents of SPUA. It provides a simple, cost-efficient, and environmentally friendly method to obtain hydrophobic cotton textiles with tremendous potential industrial applications such as in waterproofing and oil/water separation properties, among others.

## Figures and Tables

**Table 1:** The recipes for the preparation of polyurethane acrylate (PUA) and silicone modified PUA (SPUA)I. MI, multi-hydroxylalkyl silicones; TEG, triethylene glycol; PPG1000, polypropylene glycol; HEA, 2-hydroxyethyl acrylate; HMDI, dicyclohexylmethane-4, 4′-diisocyanate.

Sample	MI (wt %)	HMDI (g)	PPG1000 (g)	TEG (g)	MI (g)	EA (g)	HEA (g)
PUA	0	13.10	10.00	4.5	-	40.00	2.40
SPUAI-5 *	5	13.10	10.00	4.27	1.57	40.00	2.40
SPUAI-10 *	10	13.10	10.00	4.03	3.28	40.00	2.40
SPUAI-20 *	20	13.10	10.00	3.46	7.24	40.00	2.40
SPUAI-30 *	30	13.10	10.00	2.75	12.11	40.00	2.40

* The number means the corresponding weight percent of MI in SPUA prepolymers.

**Table 2:** Surface silicon contents, thermal analysis, and WCA data for PUA and SPUA films.

Samples	Si Content ^a^ (wt%) Measured Caculated		F ^b^	T_on_^c^ (°C)	T_50_^d^ (°C)	T_g_^e^ (°C)	WCA ^f^ (°)
**PUA**	0.25	0	-	287	355	−17.1	79.9 (109.7)
SPUAI-5	3.21	1.04	3.09	290	359	−18.9	110.1 (130.2)
SPUAI-10	4.61	2.09	2.21	289	359	−20.0	101.1 (134.0)
SPUAI-20	8.05	4.17	1.93	289	358	−22.9	103.3 (133.3)
SPUAI-30	12.12	6.26	1.93	274	358	−27.3	103.4 (129.5)
SPUAII-5	2.90	1.02	2.84	296	361	−18.5	107.4 (138.5)
SPUAII-10	4.38	2.05	2.14	292	361	−18.6	104.6 (126.5)
SPUAII-20	8.63	4.10	2.10	287	358	−18.9	103.4 (122.1)
SPUAII-30	12.03	6.15	1.96	286	356	−19.4	102.2 (130.6)
SPUAIII-5	2.24	1.05	2.13	295	367	−16.8	107.4 (129.0)
SPUAIII-10	4.28	2.10	2.04	288	360	−13.3	107.2 (124.1)
SPUAIII-20	8.04	4.20	1.91	288	359	−14.4	110.1 (132.9)
SPUAIII-30	10.05	6.30	1.59	292	360	−17.3	106.0 (129.8)

^a^ Si content, the silicon atom content of the film surface, the measured values are measured by EDS analysis, and the calculated values are the average silicon contents from the recipes of the preparation. ^b^ F is the silicon enrichment factor, F = (measured wt% of silicon) / (calculated wt% of silicon). ^c^ T_on_, the onset degradation temperature, refers to the temperature of 5% weight loss. ^d^ T_50_ refers to the temperature of 50% weight loss. ^e^ T_g_, the glass transition temperature. ^f^ WCA, the water contact angle of films and treated cotton textiles (in parentheses).

## Supplementary Materials

The following are available online at https://www.mdpi.com/2073-4360/12/8/1629/s1, Figure S1: FTIR Spectra of PUA, SPUAI (a), SPUAII (b) and SPUAIII (c) prepolymers, Table S1: The recipes for PUA and SPUAI-III with different silicone contents.
