# Supplementary material for: Preparation and Characterization of Tris(trimethylsiloxy)silyl Modified Polyurethane Acrylates and Their Application in Textile Treatment
Source: Polymers (Basel). 2020 Jul 22;12(8):1629. doi: 10.3390/polym12081629 (PMC7463466; doi:10.3390/polym12081629)
Supplement: Supplementary file 1 [file polymers-12-01629-s001.pdf]

Supplementary Materials

Preparation and Characterization of Tris(trimethylsiloxy)silyl Modified Polyurethane Acrylates and their Application in Textile Treatment

Xuecheng Yu, Ying Xiong, Zhen Li, Hongding Tang*

Engineering Research Center of Organosilicon Compounds & Materials, Ministry of Education, College of Chemistry and Molecular Sciences, Wuhan University, Wuhan, 430072, P. R. China.

E-mail address: chhdtang@whu.edu.cn (H. Tang)

Table S1. The Recipes for PUA and SPUAI-III with Different Silicone Contents

Sample	Silicone Content (wt %)	HMDI (g)	PPG1000 (g)	EA (g)	TEG (g)	MI-III (g)	HEA (g)
**PUA**	0	13.10	10.00	40.00	4.5	-	2.40
**SPUAI-5**	5	13.10	10.00	40.00	4.27	1.57	2.40
**SPUAI-10**	10	13.10	10.00	40.00	4.03	3.28	2.40
**SPUAI-20**	20	13.10	10.00	40.00	3.46	7.24	2.40
**SPUAI-30**	30	13.10	10.00	40.00	2.75	12.11	2.40
**SPUAII-5**	5	13.10	10.00	40.00	4.29	1.56	2.40
**SPUAII-10**	10	13.10	10.00	40.00	4.05	3.28	2.40
**SPUAII-20**	20	13.10	10.00	40.00	3.51	7.25	2.40
**SPUAII-30**	30	13.10	10.00	40.00	2.84	12.15	2.40
**SPUAIII-5**	5	13.10	10.00	40.00	4.28	1.57	2.40
**SPUAIII-10**	10	13.10	10.00	40.00	4.04	3.28	2.40
**SPUAIII-20**	20	13.10	10.00	40.00	3.49	7.25	2.40
**SPUAIII-30**	30	13.10	10.00	40.00	2.80	12.13	2.40

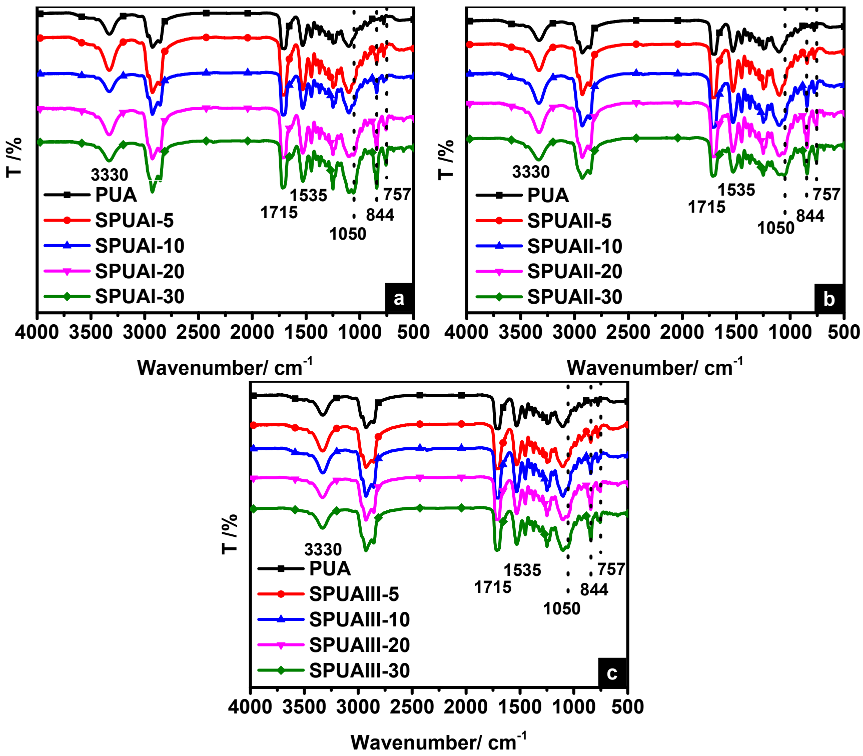

Figure S1 FTIR Spectra of PUA, SPUAI (a), SPUAII (b) and SPUAIII (c) prepolymers

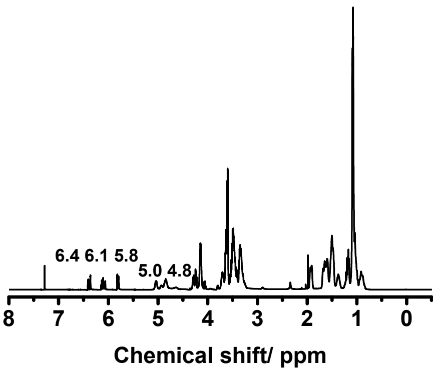

Figure S2. ^{1}H-NMR spectrum of PUA

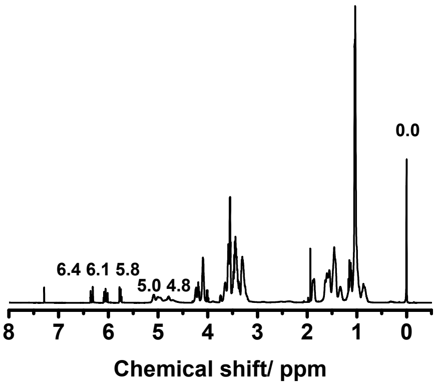

Figure S3. ^{1}H-NMR spectrum of SPUAI-5

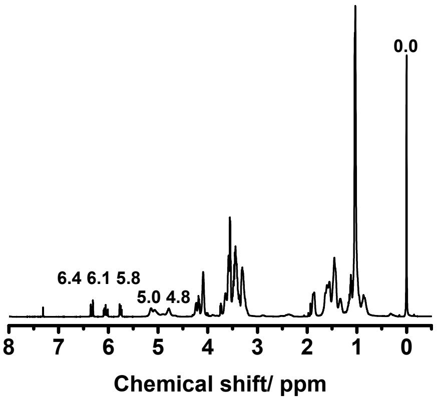

Figure S4. ^{1}H-NMR spectrum of SPUAI-10

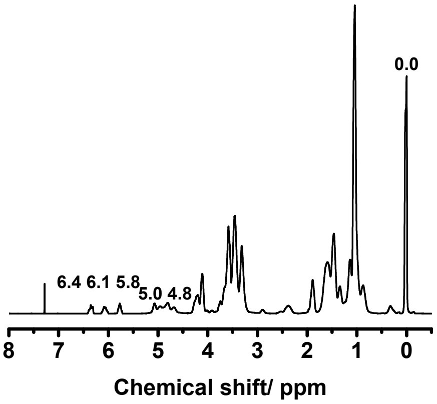

Figure S5. ^{1}H-NMR spectrum of SPUAI-20

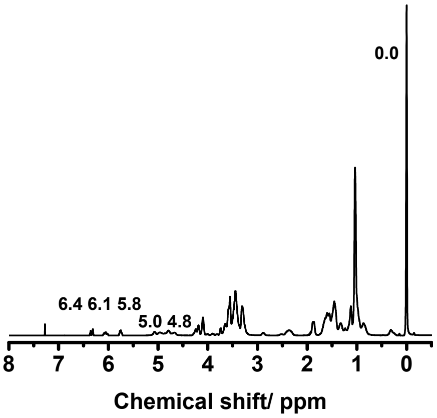

Figure S6. ^{1}H-NMR spectrum of SPUAI-30

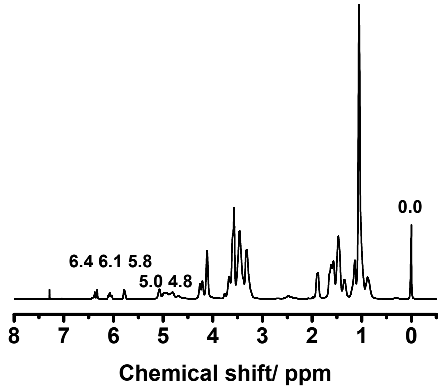

Figure S7. ^{1}H-NMR spectrum of SPUAII-5

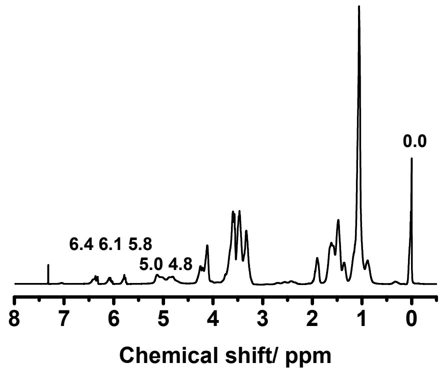

Figure S8. ^{1}H-NMR spectrum of SPUAII-10

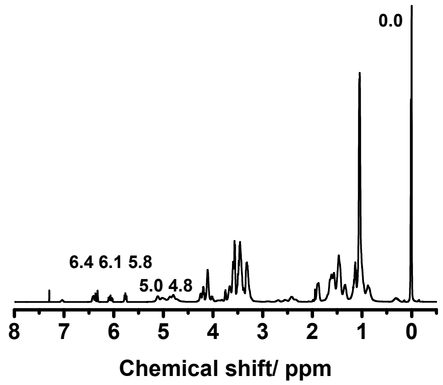

Figure S9. ^{1}H-NMR spectrum of SPUAII-20

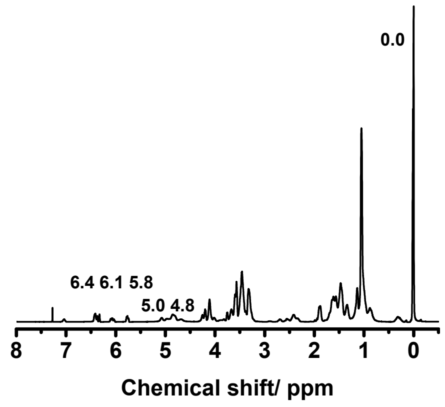

Figure S10. ^{1}H-NMR spectrum of SPUAII-30

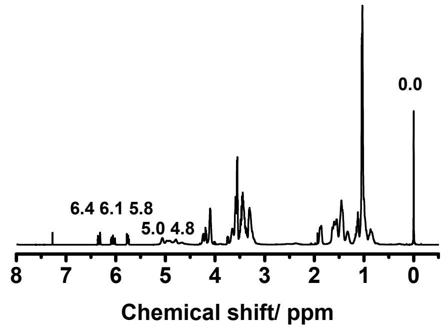

Figure S11. ^{1}H-NMR spectrum of SPUAIII-5

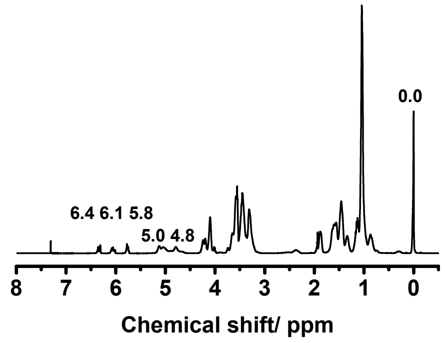

Figure S12. ^{1}H-NMR spectrum of SPUAIII-10

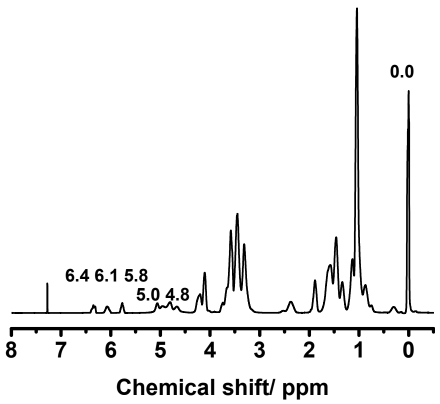

Figure S13. ^{1}H-NMR spectrum of SPUAIII-20

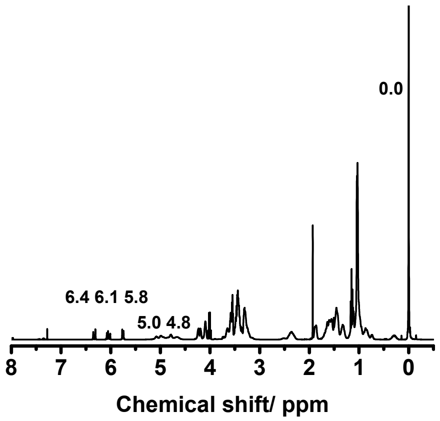

Figure S14. ^{1}H-NMR spectrum of SPUAIII-30

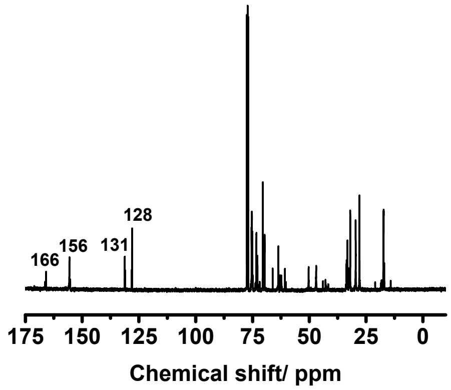

Figure S15. ^{13}C-NMR spectrum of PUA

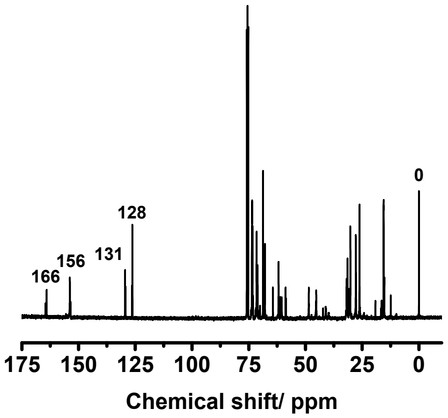

Figure S16. ^{13}C-NMR spectrum of SPUAI-5

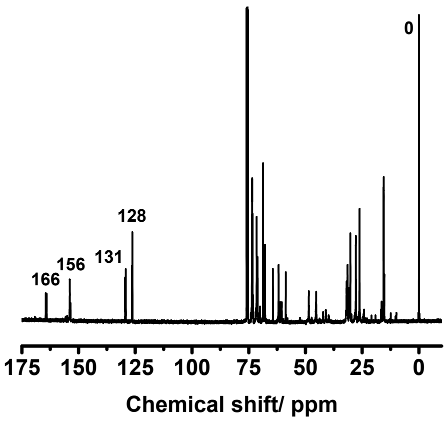

Figure S17. ^{13}C-NMR spectrum of SPUAI-10

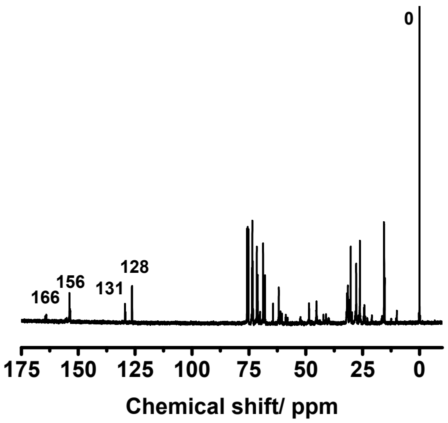

Figure S18. ^{13}C-NMR spectrum of SPUAI-20

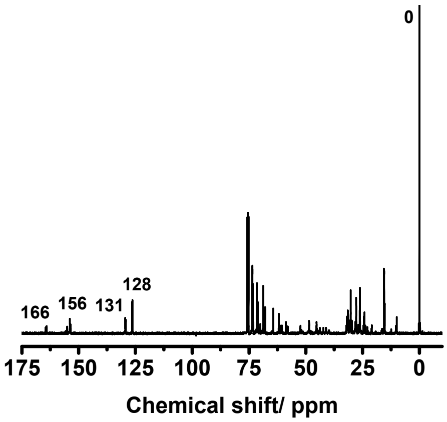

Figure S19. ^{13}C-NMR spectrum of SPUAI-30

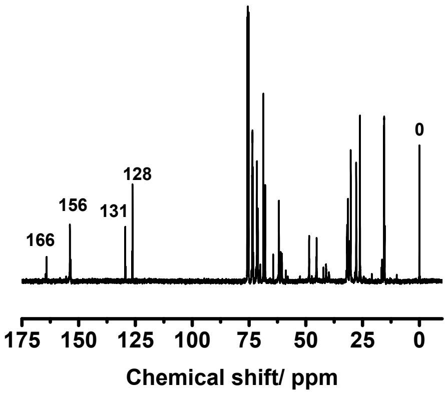

Figure S20. ^{13}C-NMR spectrum of SPUAII-5

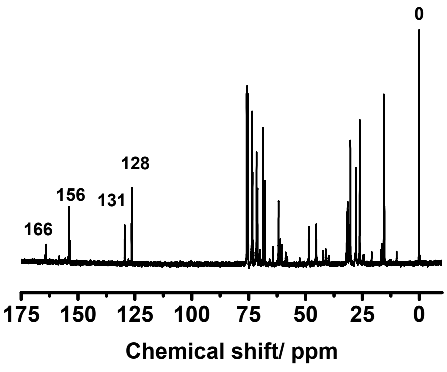

Figure S21. ^{13}C-NMR spectrum of SPUAII-10

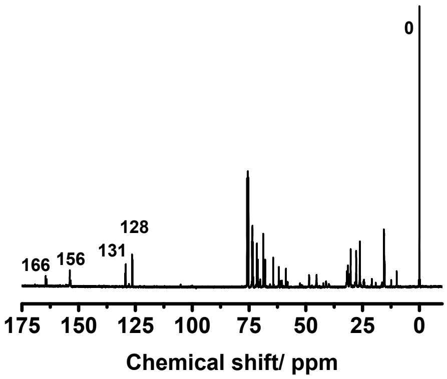

Figure S22. ^{13}C-NMR spectrum of SPUAII-20

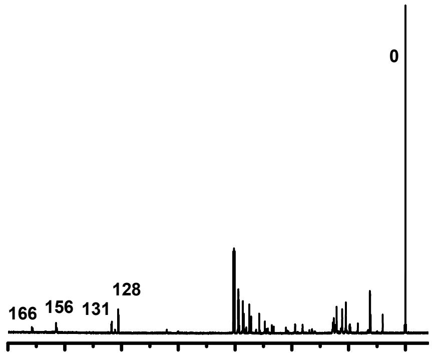

Figure S23. ^{13}C-NMR spectrum of SPUAII-30

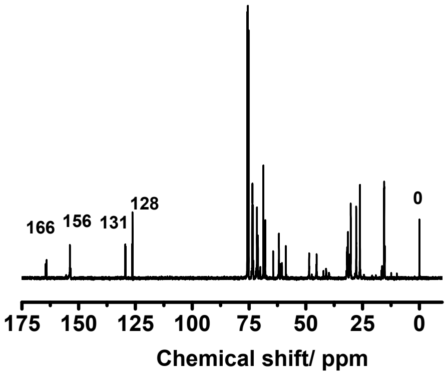

Figure S24. ^{13}C-NMR spectrum of SPUAIII-5

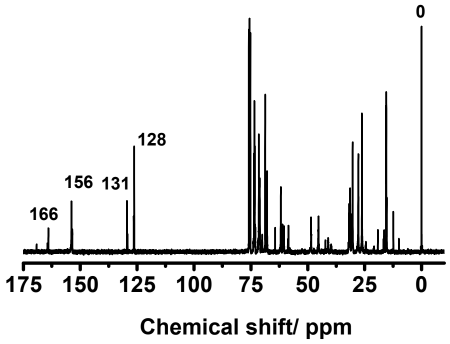

Figure S25. ^{13}C-NMR spectrum of SPUAIII-10

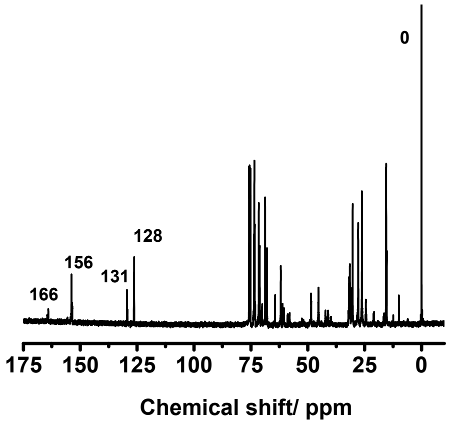

Figure S26. ^{13}C-NMR spectrum of SPUAIII-20

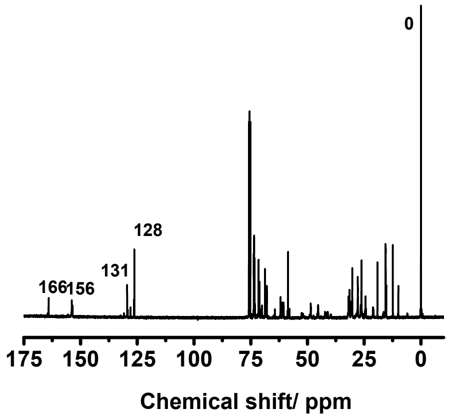

Figure S27. ^{13}C-NMR spectrum of SPUAIII-30

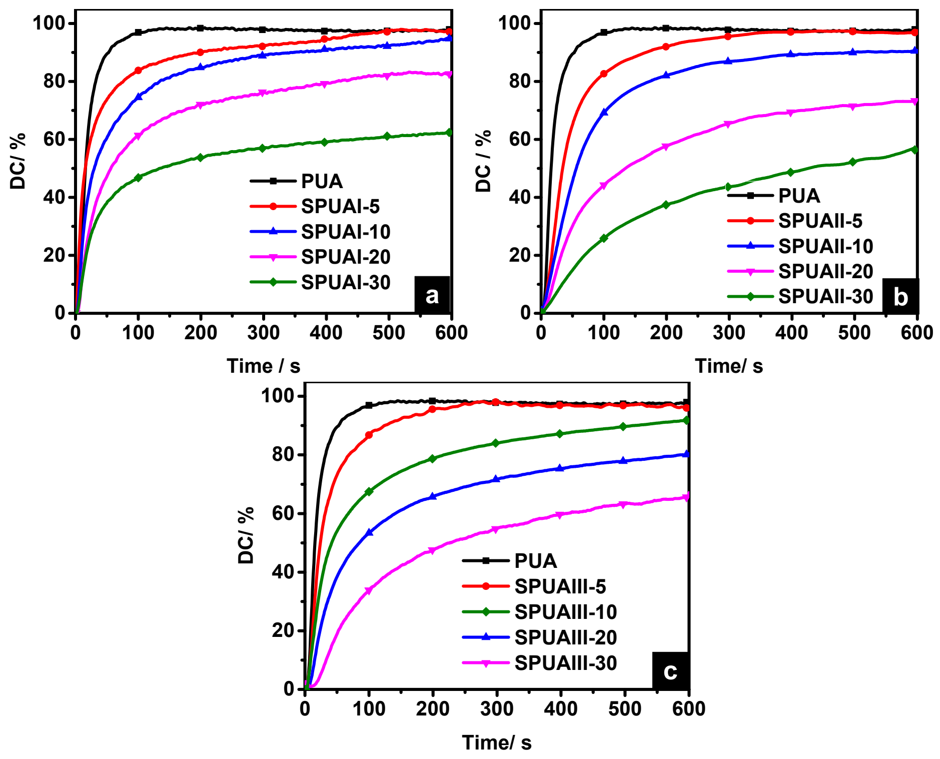

Figure S28. Photopolymerization profiles of PUA, SPUAI (a), SPUAII (b) and SPUAIII (c) prepolymers with 1.00 wt% of HMPP

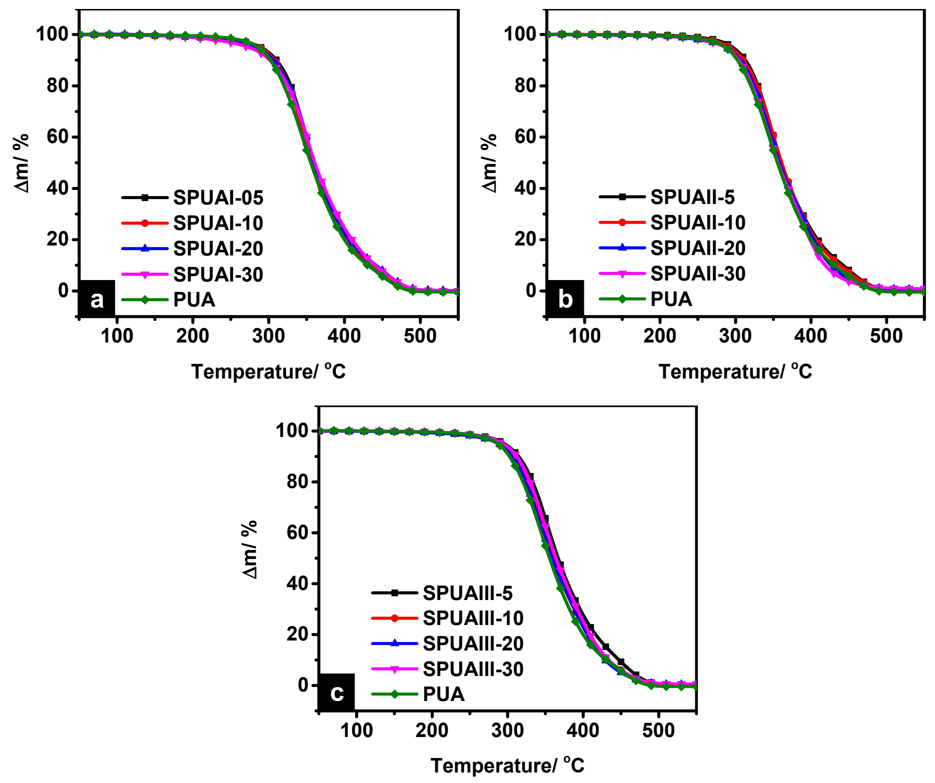

Figure S29. TGA curves for PUA, SPUAI (a), SPUAII (b) and SPUAIII (c) films

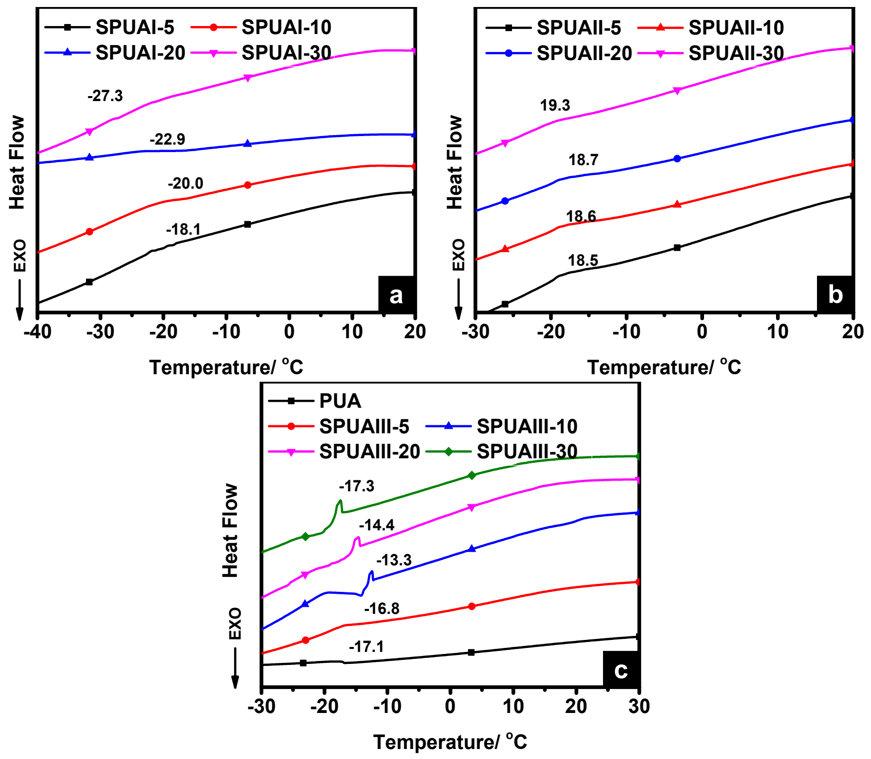

Figure S30. DSC spectrums of PUA, SPUAI (a), SPUAII (b) and SPUAIII (c) films
